# Comparison of Anterior and Lateral Approach in Hip Hemiarthroplasty for Femur Neck Fractures in the Elderly: Clinical and Radiographic Outcomes

**DOI:** 10.5704/MOJ.2211.017

**Published:** 2022-11

**Authors:** M Faggiani, S Risitano, L Rissolio, C Baroni, F Alberghina, L Conforti

**Affiliations:** Department of Orthopaedics and Traumatology, ASL TO 5, Turin, Italy

**Keywords:** hemiarthroplasty, direct anterior approach, femur neck fractures

## Abstract

**Introduction:**

Many surgical approaches have been described for hip hemiarthroplasty (HHA) treating femur neck fractures (FNFs). Direct lateral approach (DLA) is one of the most used. Today, the direct anterior approach (DAA) has become very attractive, but it seems to involve more intra-operative fractures. Our main endpoint was to demonstrate that the DAA may be a valid alternative comparing to the DLA.

**Materials and methods:**

Patients affected by FNFs and treated with HHA between the years 2016 and 2020 were studied. We divided the treatment of the fractures according to the surgical approach. The analysis was focused on perioperative complications and radiological outcomes.

**Results:**

There were a total of 166 patients. The DLA group included patients with an average age of 83.5 years and the DAA group of 83 years. We found similar surgical times (DLA 67 min vs DAA 61 min; p = 0,55), number of transfusions (DLA 3/person vs DAA 4/person; p = 0,91), perioperative complications (fractures: DLA 0 vs DAA 0 – dislocations: DLA 2,50% vs DAA 0) and functional outcomes (HHS: DLA 83 points vs DAA 87 points; p = 0,71). There were no statistical differences comparing diaphyseal filling (Canal Fill Index at the proximal third: DLA 0,79 vs DAA 0,78; p= 0,24), bone loss (Paprosky I: DLA 96,25% vs DAA 91,86%; p = 0,47) and prevalence of heterotopic ossification (Broker low degree: DLA 93,75% vs 95, 34%; p = 0,87).

**Conclusion:**

Analysing perioperative complications and studying post-operative radiographic evolution, our results suggest that the DAA is a valid alternative to the DLA in HHA treating FNFs.

## Introduction

Fractures of the proximal femur are among the most common injuries in elderly patients1. Femoral neck fractures (FNFs) constitute more than half of these fractures, with displacement of the femoral head fragment in nearly 70% of cases^2^. In these patients, surgery is generally required^3^. As safe and relatively short-time procedure, hip hemiarthroplasty (HHA) is frequently performed^4^. Currently one of the most used approaches for HHA is the direct lateral approach (DLA)^5^. The DLA provides excellent exposure of both proximal femur and acetabulum but requires partial separation or retraction of the insertion of the gluteus medius muscle for an adequate exposure of the capsule^5^. The good exposure of the components helps in the correct choice of the femoral stem and potentially reduces the subsequent reduction of bone damage. However, such exposure may cause an area of reduced resistance that can determine easier episodes of dislocation and a post-operative abductor muscle dysfunction^1,5^. Interest for direct anterior approach (DAA) has greatly increased in recent years for elective total hip arthroplasty (THA)^6-8^. The DAA approach consists of a muscle-sparing and inter-nervous approach, which offers a potentially less invasive surgery^9^. The literature supports its association with reduced risk of dislocation compared to other surgical approaches, however some studies have reported increased surgical time and rate of intra-operative femur fractures (IFFs)^9,10^. Although outcomes of these approaches have been compared in numerous studies, the best surgical approach for hip hemi-replacement in femoral neck fractures (FNFs) has not been clearly identified^11-14^.

Is the DAA statistically different from the DLA? Our main endpoint was to demonstrate that the DAA may be a valid alternative compared to the DLA, without a lengthening of surgical times or an increase of the perioperative complications and then reject our zero hypothesis. Compared to the previous literature, our analysis does not only focus on clinical and functional results, but a careful analysis of the radiographic data was also carried out^11-13^.

## Materials and Methods

From November 2016 to November 2020 a case series study was undertaken in our Orthopaedic and Traumatology Complex Structure in a Local Health Department (Azienda Sanitaria Locale, ASL) of Turin (Italy). The inclusion criteria were as follow: HHA because of FNFs (AO/OTA 31 A1, B1-B3) in patients older than 65 years. Patients were excluded if they suffered from pathological or other associated fractures. A single independent investigator (VP), who was not involved in the treatment of the patients, randomly allocated participants to either the DLA versus the DAA by computer-generated randomisation. All surgical procedures were performed with the same implant (Gladiator Bipolar System) and by the same surgical team composed of three orthopaedic specialists experienced in both approaches. The instruments available were specific for the anterior or the lateral approach. The choice of cementation was made at the surgical time: the stem in subjects with good bone tissue was not cemented. The Institutional Review Board of our institution approved this study. Basic data (age, gender, and demographic parameters), clinical details (comorbidities, ASA score, fractures, and surgical treatment characteristics) and radiographic imaging (anteroposterior hip/pelvis and lateral hip radiographs) were analysed retrospectively collecting the data from the hospital database by a second external investigator (VP2). We subdivided the analysis in the perioperative time (T0), post-operative time (T1), at 3 months from the surgery (T2) and at the last follow-up (12 months, T3), and we focused the analysis on the following variables: mean surgical time (from skin incision to the final suture)^14^; number of post-treatment transfusions (our anaesthesiologist’s protocol recommends transfusion of two bags of hematite’s with iron concentration of 25mg per 250cc bag for all patients with cardiological pathology diagnosed by clinical and instrumental examinations, an diagnosed before trauma with haemoglobin of less than 10g/dl); perioperative or post-operative periprosthetic fractures (Vancouver Classification)^15,16^; post-operative episodes of dislocation; the average of post-operative Canal Fill Index (CFI) (diaphyseal filling calculated anteroposterior hip radiograph at the superior-third juncture, mid-third and distal extremity of the stem)^17^; evaluation of the post-treatment functional recovery calculating the Harris Hip Score at T318; metadiaphyseal bone loss (from Grade I to IV; Paprosky classification) at T319; the extent of heterotopic ossification (HO) (low, moderate, and high; simplified Broker classification) from T0 to T3. The protocol on HO prevention is the administration of celecoxib 200mg 1 tablet twice a day for 21 days^20^.

Post-operative treatment was performed according to the same rehabilitation protocol. After about 7-15 days patients were sent to the external rehabilitation centre. Our goal was to reach a sample of at least 150 patients with a follow-up of 6 months and to compare the surgical variables (surgical time and intra-operative fractures) and the post-operative outcomes (transfusions numbers, functional and results from radiographs).

Statistical analysis was performed using the SPSS version 15.0 software [SPSS Inc, Chicago, IL, USA]. The central trend measures (average and statistical mode) and the dispersion measures (standard deviation, DS) were calculated for numerical values. The categorical variables were expressed in percentage. The Student T Test was used to compare continuous variables, while the Chi Square Test was applied to evaluate the relationship between categorical data. Ordinal variables were compared using the Mann Whitney U-test. Statistically significant result for values of p<0,05 were considered relevant. The level of significance was fixed at 5%, with a power of 85%, and the calculated sample number corresponded to 68 patients per group.

The present study was conducted in accordance with the ethical standards laid down in the 1964 Declaration of Helsinki and the informed consent for processing of data was obtained from all patients when admitted to the hospital.

## Results

The initial cohort was composed of 207 patients with FNFs. After inclusion and exclusion criteria were applied, there were 166 patients identified for the study. A percentage of 45.05% (53 patients) showed more than 2 relevant comorbidities at T0. During the pre-operative anaesthesiology evaluation, the ASA score was found to be more than Grade 2 in 38.03% (32 subjects) of cases. According to the initial randomisation, the sample was divided in the DLA and DAA group. The DLA group included 80 patients (33.75% males and 66.25% females) with a mean age of 83.5 years (range 75-95). Based on radiographs performed at T0, 93.75% (75 patients) were characterised by an AO/OTA 31B1-B3 fractures, the others 6.25% (5 cases) by an AO/OTA 31A1. Most of the subjects (75 cases; 93.75%) were treated with a cemented implant. The DAA group consisted of 86 cases (25.58% males and 75.58% females) with an average age of 83 years (range 66102). A total of 97,67% (84 patients) were AO/OTA 31B1-B3 fractures, the others 2.33% (2 cases) AO/OTA 31A. A total of 89.53% (77 patients) HHAs featured a cemented implant.

The results showed normal distribution and no significant difference between the groups (p=0.35) and fractures characteristics (p=0,55). We have reached an average of follow-up of 12 months for each group (DLA 5-15, loss to follow-up 1.2%; DAA 6-18, loss to follow-up 1.4%) (Table I). The first group had an average length of stay of 10 days (7-18) and the second of 12 days (5-21). The average of the surgical time was similar between the two groups: 67 min (range 30-90) through the lateral approach comparing to 61 min (range 35-85) for the anterior (p=0,87). Very similar number of transfusions were carried out during the recovery time, without significant differences (DLA average 3/person; range 0-6. DAA average 4/person; 0-5. p=0,91) (Table II). We reported two cases of dislocation in the DLA group (2.50%), and one (1.16%) in the DAA. We have reported no case of fracture at the intra-operative time.

**Table I:** Demographic and clinical sample characteristics

	DLA (range; %)	DAA (range; %)
Patients	80	86
Sex (male : female)	27:53 (33.75 : 66.25)	21:65 (25.58 : 75.58)
Age	83.5 (75-95)	83 (66-102)
AO/OTA		
31B1-B3	75 (93, 75)	84 (97, 67)
31A1	5 (6, 25)	2 (2, 33)
HHA		
cemented	75 (96, 25)	77 (89, 53)
Non-cemented	5 (6, 25)	9 (10, 47)

**Table II:** Results. (HHS: Harris Hip Score, CFI: Canal Fill Index, HO: Heterotopic Ossification)

	DLA (range; %)	DAA (range; %)	p value
Surgical time	67 (30-90)	61 (35-85)	p=0.87
Transfusions	3/person (0-6)	4/person (0-5)	p=0.91
Dislocations	2 (2.50)	0	p=0.97
Periprosthetic fractures	0	0	
HHS	83±13.4	87±14.1	p=0.71
CFI		p=0.24	
Proximal third	0.79 (0.75 - 0.80)	0.78 (0.74 - 0.81)	
Medial third	0.80 (0.75 - 0.83)	0.73 (0.71 - 0.75)	
Distal third	0.62 (0.59, 0.65)	0.61 (0.57 - 0.68)	
Bone loss			p=0.47
First degree	77 (96, 25)	79 (91, 86)	
Second degree	3 (2, 4)	7 (8, 13)	
HO		p=0.87	
Low degree	75 (93, 75)	82 (95, 34)	
High degree	2 (2, 5)	4 (4, 65)	

**Fig. 1.**
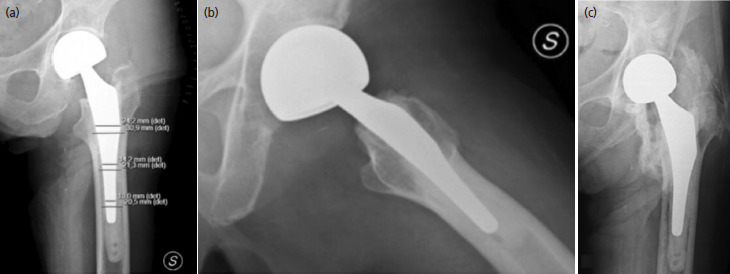
(a) Anteroposterior radiography after hip-hemiarthroplasty through the lateral approach. Example of Canal Fill Index calculation. (b) Post-operative lateral view. (c) Example of heterotopic ossification (Grade 4, second Brooker Classification) 12 months after surgery.

**Fig. 2.**
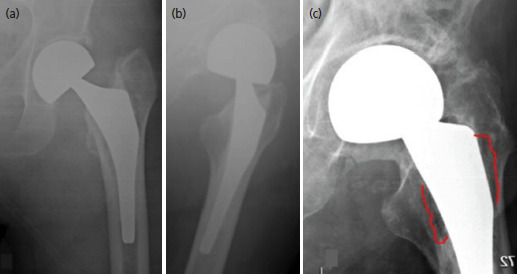
(a) Left hip radiographic anteroposterior view after hemiarthroplasty through anterior approach. (b) Post-operative lateral view. (c) Area of bone resorption (red line) and heterotopic ossification 12 months after surgery.

During the anterior, at each surgical step, the levers have been relocated until the correct view of all joint components, this precaution has probably allowed a reduction of the intra-operative fractures. At T3, the average HHS was 83±13,4 for patients treated with the lateral approach compared to 87±14,1 for the DAA group (DS 2.67; p=0.71) (Table II), without no Trendelenburg sign. Our results showed that the diaphyseal filling of the stem was 0.79 (0.75-0.80) vs 0.78 (0.74-0.81) at the proximal third, 0.80 (0.75-0.83) vs 0.73 (0.71-0.75) at the medial and 0.62 (0.59-0.65) vs 0.61(0.570.68) at the distal third in DLA and DAA group, respectively (Fig. 1). The comparison between the data showed higher filling in DLA group at the medial third, without statistical relevance (p=0.24). Based on Paprosky classification, the analysis of the imaging at the T3 showed a similar bone defect between the samples: 77 (96.25% of DLA) vs 79 patients (91.86% of DAA) of first degree, and just 3 (2.4%) vs 5 patients (8.13%) of second (p=0.47) (Fig. 2, Table II). Analysing the pelvis/hip radiographs at T0 and T3, we showed that the surgical approach did not significantly affect the onset of HO (p=0.87). According to the simplified Broker classification, we proved that most of the sample developed a low degree of HO (93.75% of DLA group and 95.34% of DAA). Subjects who developed a high degree of ossification were only the 2.50% in the DLA group (Fig. 1) and 7.65% in the DAA (Table II). From the results obtained it is possible to affirm that there is no functional or radiographic significant difference between the two techniques.

## Discussion

Many surgical approaches have been described for hip replacement arthroplasty in trauma patients^21^. DLA is one of the most used providing excellent exposure of both the proximal femur and acetabulum^1^. However, several authors reported as a partial dissection of the gluteus medius muscle insertion for an adequate exposure of the capsule might affect a post-operative abductor muscle function^12,14,18^. In recent years, the anterior surgical approach has become very attractive for many hip surgeons^1^. This procedure has been diffused in the surgical practice of the total hip arthroplasty (THA)^5^. The literature shows that the DAA leads to a less invasive approach, low dislocation rates (preserving the external rotators muscles with a potential increasing of postoperative stability) and intact abductor function^5,6,10^. In terms of complications, the DAA demonstrates more intra-operative periprosthetic fractures with an incidence about of 2.3% and the higher risk of femoral intra-operative fracture led many surgeons to switch their own preference on posterior or lateral approach treating hip fractures in older patients. Few studies on the DAA analysed elderly populations affected by FNFs^10,22^, however, the correlation between fracture risk, cementation, stem length and size are still unclear^3,15^. Other research compared the functional outcomes resulting from the implantation of endoprosthesis through the anterior and lateral approach^11-13,23^, but there is no detailed analysis of the radiographic results. Hip fractures in the elderly represent a major public health burden worldwide and the identification of the best approach could lead to faster rehabilitation and better functional outcomes in this fragile population^3,24^.

In our study, the main endpoint was to demonstrate that the DAA may be a valid alternative comparing to the DLA for HHA in the treatment of FNFs, without a lengthening of surgical times or an increase of the perioperative complications. Differently from previous studies, our analysis did not focus exclusively on the comparison of clinical data such as surgical time, blood loss counts and perioperative complications, functional parameters, but also considered the radiographic characteristics such as the diaphyseal stem filling (CFI), the metadiaphiseal bone loss (Paprosky classification) and the extension of the HO (Broker classification)^19^. Taking a sample of people over 65 years old, we divided the femur fractures according to the surgical approach carried out for the HHA implantation. Comparing the basic data of the DLA and the DAA group, the two populations were comparable (number of patients, sex, age, and kind of fractures; p=0,95). We highlighted a similar average of surgical time between the two groups (67 vs 61 min) with a reduced timing for the anterior approach; we reached the same conclusions as the study of Neyiscy *et al* and Carlson *et al*^12,25^ without, however, identifying a significant difference (p=0.55). We did not experience a surgical learning difficulty, as indicated in other studies^10,22^. The comparison of blood loss among studies is difficult but, compared to other studies^2,12^, the number of transfusions does not differ significantly between the groups (p=0.12). Despite the lateral approach having been more frequently associated with dislocations compared to the anterior approach^4,5,10^, in our study there was a no significant tendency towards a higher risk of dislocations after the lateral approach (p=0,97; probably determined by an accurate surgical closure of the joint capsule). According to Van der Sijp *et al* (2018)^1^, despite the higher age in our sample (average of 87 years old, 72-98), we have found that the intra-operative fractures were not related to the approach type (p=0.91). Similar to this present study, there are some current Cochrane reviews, meta-analyses and other research work that compared perioperative complications between the two approaches in FNF patients, without detecting significant differences^24,26^. Disagreeing with Lakhani *et al* (2022) that identified best functional results after the anterior approach^23^, at 3 months of follow-up (Time 2) the average HHS was 83±13,4 for DLA group compared to 87±14,1 for the DAA (DS 2.67; p=0.71) without a real differences between the two cohort and no Trendelenburg sign. The choice of the femoral component size is the principal cause of the femoral component filling^17^. Dietrich *et al* and McCloskey *et al* have shown that an inadequate stem size could lead to an increased risk of intra-operative fracture^8,15^. Only the Langlois *et al* study analysed the radiographic outcomes by comparing the anterior approach with the lateral one, analysing the alignment and the offset^24^.

In our data, the CFI revealed that the lateral approach seems to allow a better filling of the stem in the middle third (p=0.24). According to the previous analysis on total hip arthroplasty, this result could be explained by a better exposure of the components through the lateral approach^5^. The exposure of the components and the correct choice of the stem size results in a reduction of the bone defect progression^10^ and therefore in reduction of subsequent mobilisation. If the proximal metaphyseal bone is damaged, it may not be mechanically supportive for a proximally fitting implant^19^. Comparing these two approaches, in literature there is no study regarding the progression of the bone loss. In our study there was no statistical differences between cohorts in radiographic signs of instability, migration of the stem and progressive radiolucent lines (p=0.47). Investigating the prevalence of heterotopic ossification (HO) following direct anterior approach in total hip arthroplasty, Van der Sijp *et al* found a rate of 37.1%^13^. The literature hypothesised that trauma to the soft tissues might be an inciting event for HO^10,12,20^. This assumption was refuted by our calculations, we showed that the surgical approach did not significantly affect the onset of HO (p=0.87). As indicated in some studies cited earlier^12,13,19,20^, HO should be less associated with the anterior approach, as should the progression of bone loss in the lateral approach. Our study did not identify any radiographically significant difference between the two techniques. For example, we have reported cases of progression towards the formation of HO in both approaches in Fig. 1 and 2. The data obtained do not allow us to identify a better approach than another. Our research can direct the surgical team to perform daily both approaches in the femur fractures of the elderly, without running into increased risks of perioperative complications or worse functional and radiographic results.

The study has limits: the sample number is small with a reduced analytical power, the surgeons and the VP2 were aware of the type of surgical approach undertaken, the analysis of the data examined were retrospective, the follow-up is short, the infection rate and mortality are relevant parameters but not considered for the presence of errors in the stratification of associated comorbidities.

## Conclusion

The study of the perioperative variables considered (high surgical time, number of transfusions, fractures, dislocations, and functional recovery) and the post-operative radiographic evolution (CFI, bone loss and HO extension) suggest that the DAA may be a valid alternative to the DLA in HHA in FNFs treatment. We need studies with larger samples and analysis of relevant variables such as infection rate and mortality to identify the gold standard approach.
